# Selected Molecular Mechanisms Involved in the Parasite–Host System *Hymenolepis diminuta–Rattus norvegicus*

**DOI:** 10.3390/ijms19082435

**Published:** 2018-08-17

**Authors:** Patrycja Kapczuk, Danuta Kosik-Bogacka, Natalia Łanocha-Arendarczyk, Izabela Gutowska, Patrycja Kupnicka, Dariusz Chlubek, Irena Baranowska-Bosiacka

**Affiliations:** 1 Department of Biochemistry and Medical Chemistry, Pomeranian Medical University, Powstańców Wlkp. 72, 70-111 Szczecin, Poland; patrycja2510@o2.pl (P.K.); patrycjakupnicka@o2.pl (P.K.); dchlubek@sci.pam.szczecin.pl (D.C.); 2 Department of Biology and Medical Parasitology, Pomeranian Medical University, Powstańców Wlkp. 72, 70-111 Szczecin, Poland; kodan@pum.edu.pl (D.K.-B.); natalia.lanocha@pum.edu.pl (N.Ł.-A.); 3 Department of Biochemistry and Human Nutrition, Pomeranian Medical University, Broniewskiego 24, 71-460 Szczecin, Poland; izagut@poczta.onet.pl

**Keywords:** hymenolepidosis, molecular mechanisms, parasite–host system, rat

## Abstract

The rat tapeworm *Hymenolepis diminuta* is a parasite of the small intestine of rodents (mainly mice and rats), and accidentally humans. It is classified as a non-invasive tapeworm due to the lack of hooks on the tapeworm’s scolex, which could cause mechanical damage to host tissues. However, many studies have shown that metabolites secreted by *H. diminuta* interfere with the functioning of the host’s gastrointestinal tract, causing an increase in salivary secretion, suppression of gastric acid secretion, and an increase in the trypsin activity in the duodenum chyme. Our work presents the biochemical and molecular mechanisms of a parasite-host interaction, including the influence on ion transport and host intestinal microflora, morphology and biochemical parameters of blood, secretion of antioxidant enzymes, expression of Toll-like receptors, mechanisms of immune response, as well as the expression and activity of cyclooxygenases. We emphasize the interrelations between the parasite and the host at the cellular level resulting from the direct impact of the parasite as well as host defense reactions that lead to changes in the host’s tissues and organs.

## 1. Introduction

Over the course of evolution, parasitic organisms and their hosts have developed mutual morphological, physiological, and biological relationships that have led to the formation of parasite-host systems [1]. In most cases, parasitic infections, especially by helminths, are asymptomatic and have low intensity. However, the interactions between the parasites and their hosts may sometimes lead to changes in the host tissues and organs, resulting both from the direct effect of the parasite and induced defense mechanisms. For example, the hooks of the pork tapeworm (*Taenia solium*) cause damage to the intestinal epithelium, and large helminths can cause obstruction of the intestinal lumen (e.g., *Diphyllobothriid* family cestodes, *Ascaris lumbricoides*), as well as obstruction of bile ducts (*Fasciola hepatica*) and lymphatic vessels (*Wuchereria bancrofti*) [2]. The presence of larvae of *Echinococcus granulosus* in the liver results in pressure and disturbances in the proper functioning of this organ. Finally, different metabolites secreted by parasites may also affect the functioning of the host organism. Proteolytic enzymes, including acetylcholine and histamine secreted by *Enterobius vermicularis* and *F. hepatica,* cause inflammation, inhibition of blood coagulation and dilation of blood vessels [2,3,4,5].

The interactions between the parasite and the host have an effect on the immune response of the host. Occasionally, there is an excessive and inadequately directed host defense response leading to the degradation of its tissues and organs [2,6]. The immunopathological reactions result in changes in the host organism, including enlargement of internal organs (spleen, liver, lymph nodes) due to the increased activity of immune cells (macrophages and lymphocytes), inflammatory lesions and foci in the liver (cysts, abscesses, granulomas), immune complexes in the kidneys, and the anaphylactic reaction and shock [7,8,9].

Parasitic infections activate all kinds of non-specific (innate) and specific (acquired) immune responses, the latter consisting of cellular and humoral components. A specific response develops during the first contact with the parasite and its antigens, followed by the stimulation of T helper cells and B lymphocytes for the production of antibodies [10]. In turn, the non-specific response is related to the occurrence of natural physico-mechanical (maintaining tissue continuity), biological (secretion of lysozyme, lactoferrin) and chemical barriers (low pH of the skin and stomach) of the host organism. However, the most characteristic phenomenon determining the non-specific response is phagocytosis, which occurs through macrophages, neutrophils, eosinophils and components of the complement system in the environment of reactive oxygen and nitrogen. A parasitic invasion triggers a humoral immune response involving the production of immunoglobulins, the control of extracellular parasites present in the blood and in bodily fluids, as well as a cellular response associated with the removal of intracellular parasites [6].

The biochemical, histochemical, and pathophysiological aspects of the parasite–host mutual relationship using various experimental models have been the subject of many studies [11,12,13,14]. However, the molecular mechanisms of these interactions are still not fully understood. In the present work, we show the biochemical and molecular mechanisms of parasite–host interaction based on the recent research on the rat tapeworm *Hymenolepis diminuta*. These include the influence on ion transport, host intestinal microflora composition, blood morphology and biochemical parameters, secretion of antioxidant enzymes, expression of Toll-like receptors, mechanisms of immune response, as well as the expression and activity of cyclooxygenases. The work emphasizes the interrelations between the parasite and host at the cellular level resulting from the direct impact of the parasite, as well as host defense reactions that lead to changes in the host’s tissues and organs.

## 2. Rat Tapeworm *Hymenolepis diminuta* Rudolphi, 1819

The rat tapeworm *Hymenolepis diminuta* (Cestoda) is an intestinal parasite of small rodents, including mice and rats. Humans are only accidental hosts [15,16,17,18,19,20]. The strobila of a mature tapeworm can reach 20 cm to 60 cm in length and 3 mm to 5 mm in width. It contains from 800 to 1000 proglottids, which are 3.5 mm wide and 0.76 mm long. The length of the tapeworm depends on the intensity of infection (crowding effect) [21,22,23,24]. The scolex of *H. diminuta* has no hooks but is equipped with four suction cups. The tapeworm’s eggs are oval, from 60 to 85 μm in diameter, devoid of polar filaments in the inner shell [17,25,26].

The life cycle of *H. diminuta* requires an intermediate arthropod host, including mealworms (*Tenebrio molitor*), flour beetle (*Tribolium confusum*, *Tribolium castaneum*, *Tribolium destructor*), rat fleas (*Xenopsylla cheopis*, *Nosopsyllus confusum*), mouse fleas (*Leptopsylla segnis*), as well as other insects, such as beetles and cockroaches [25]. After the ingestion of eggs present in rat feces by the arthropods, oncospheres are released from the eggs in the digestive tract, which penetrate the intestinal wall and develop into the cysticercoid larvae, usually over about 10–14 days [27,28]. Infection by ingestion of the infected arthropod containing cysticercoids results in the development of the adult worm in the intestine of the definitive host [29]. *H. diminuta* reaches maturity within 18 to 20 days, after which the gravid proglottids filled with eggs are excreted with the host’s feces [17,25,26].

The symptoms of hymenolepidosis (or hymenolepiasis) in humans are not very characteristic and are limited to indigestion, abdominal pain, and diarrhea, but most commonly the infection is asymptomatic [16,30,31,32,33,34]. Although an adult tapeworm does not have hooks in its structure that could damage host tissues, its metabolites may affect the functioning of the gastrointestinal tract by, inter alia, increasing the secretion of saliva, inhibiting the secretory capacity of the stomach, as well as increasing the activity of trypsin in the duodenum [35].

Rats are most commonly used in studies on hymenolepidosis. Mice are not a very suitable research model, as their immune system exhibits increased activity already during the first invasion. Research shows that at the first experimental infection of mice, after about two weeks, the development of this parasite is halted; reinfection results in its spontaneous and total expulsion. The rat immune system does not show such a significant activity at the first infection, although secondary infections in rats do result in the severe stunning of tapeworms or even are unsuccessful [36]. *H. diminuta*’s infrapopulations (10–20 specimens) persist in the rat throughout its entire life, up to 14 years, while high densities are followed by rapid expulsion of some parasites [37]. After infecting the experimental rats with 25 and 30 *H. diminuta* cysticercoids, the number is reduced by half after 6 weeks [38] and by 2/3 in the third month after infection [39]. At an even higher density of parasites in the rat intestine, after administration of 40 to 100 cysticeroids in the period from 1 to 3 months after infection, about 10 or fewer tapeworms remain [39,40,41,42]. After secondary infection by *H. diminuta*, the number of parasites decreases even faster [40].

### 2.1. Morphological and Histomorphological Changes in the Host’s Digestive Tract

Many studies on hymenolepidosis concern morphometric evaluation of the gastrointestinal mucosa [16,35,43,44,45,46]. Goswami et al. (2011) found changes in the liver, kidneys and intestine of wild and laboratory rats infected with *H. diminuta*. Detailed examination showed excessive mucus secretion and desquamation of the intestinal epithelium that may irritate the host’s intestinal mucosa through the edges of the tapeworm segments. Those authors also observed the flattening and even partial disappearance of intestinal villi and increased mucin secretion into the lumen of the intestine of the rats. In addition, infected rats were found to have a damaged gastrointestinal wall in the form of eosinophilic enteritis [16]. Similar results regarding the effect of *H. diminuta* invasion on the rat intestinal mucosa using a scanning light microscope, were presented by Martin and Holland (1984) [45]. Eosinophilic enteritis in rats infected with *H. diminuta* was also observed by Starke (2001) [46].

Kosik-Bogacka et al. (2012) showed changes in the morphology of intestinal villi and crypts in the digestive tract of Wistar rats infected with *H. diminuta*. The authors observed a reduction in villus length and crypt deepening in the duodenum and jejunum in the rats 16 days post infection with the tapeworm. However, such changes were not observed in the ileum and colon [44]. In a study by Fal and Czaplicka (1991) in Buffalo rats infected with *H. diminuta*, the largest changes occurred in the ileum, and the lowest in the duodenum [35]. Dwinell et al. (1998) observed inflammatory infiltrates and erosions reaching the muscle layer in rats at day 40 post infection with *H. diminuta* [15]. All researchers found a reduction in the length of the intestinal villi and crypt deepening in the gastrointestinal tract of rats. Slight discrepancies may result from the variation in the conditions of the experiments performed, including in the various strain of rats.

### 2.2. Pathophysiological and (Sub-)Cellular Changes in the Host’s Digestive Tract

The intensity of infection of rats by *H. diminuta* varies from 1 to 17 tapeworms in one host. It is assumed that the intensity of infection is high in the presence of more than 5 parasites [16,47,48,49,50,51]. Over the course of individual development, the tapeworm increases in size, which affects the secretion and absorption in the gut, and can also cause changes in the tissues of the digestive tract [15,43,52].

Protection of the intestines against the harmful influences of external factors, including parasites, is possible thanks to the tightness of the mucous membrane, peristaltic movements, physiological intestinal microflora, and the enteric nervous system (ENS), which are closely related and form the so-called gut–brain axis [53,54,55]. ENS is involved in the regulation of the activity of the digestive system, acting directly on the mucous membrane, muscles, and blood vessels. The proper functioning of ENS depends on the glial cell–derived neurotrophic factor (GDNF), which has many functions related to the nervous system. It is responsible for the survival and differentiation of dopaminergic neurons, the repair and growth of damaged intestinal neurons, and also strengthens the signals between neurons [56]. Recent studies have shown that it also takes part in the regeneration of the intestinal epithelium, maintaining the intestinal epithelial barrier, and reducing intestinal inflammation. In addition, GDNF may have an antiapoptotic effect in the gastrointestinal tract and participate in the signal transmission between enterocytes [57]. As GNDF affects ENS, Starke-Buzetti et al. (2008) examined the concentration of GDNF in the ileum and jejunum of rats infected by *H. diminuta*, and found an increased number of cells producing this neurotrophin. Therefore, it can be assumed that GDNF affects the intestinal nervous system and intestinal mucosa during hymenolepidosis [58].

The presence of *H. diminuta* in the rat gastrointestinal tract may also affect the contraction of smooth muscle in the intestines by modulation of muscarinic receptors (M) located in the cell membrane. These receptors are involved in a cascade of reactions that cause a contraction of the smooth muscle of the gastrointestinal tract and secretion of gastric juice. Acetylcholine stimulates receptor action by attaching to the G protein and changing its conformation. G proteins are characterized by a large polymorphism, stimulating signal transduction in cells in many ways. The main mechanism of their action is GTPase activity; i.e., hydrolysis of guanozyno-5′-trifosforan (GTP) to guanozyno-5′-difosforan (GDP). They belong to the adapter proteins for receptors located in the cell membrane, responsible for the regulation of ion channels [59]. Bikopoluos et al., (2006) investigated the effect of *H. diminuta* infection on changes in acetylcholine metabolism and expression of muscarinic mRNA in jejunum in rats. In that experiment, the expression levels of the M1, M2, and M3 receptors and acetylcholinesterase were determined. The study showed that infection with *H. diminuta* may affect the expression of the M2 receptor mRNA, and in the case of a high intensity of infection, expression of the M1 receptor mRNA is inhibited [55,60]. The observed changes in the level of neurotransmitters, secretion, and the regulation of peristalsis in the gastrointestinal tract confirm the influence of *H. diminuta* on intestinal remodeling. Changes in intestinal activity observed during infection might reflect that tapeworms may slow down intestinal peristalsis, which is used by the host to remove the parasite from the host.

### 2.3. Influence of Gastrointestinal Flora on the Course of H. Diminuta Infection

Previous studies have shown that the natural bacterial flora of the host’s digestive tract affects the course of the parasitic invasion. This dependence probably results from the fact of colonizing the same environmental niche. Intestinal parasites, including helminths, interact with the bacterial flora, which may disturb the balance in the host microflora. On the other hand, microbes of the digestive tract may also influence the course of parasitosis [61,62]. Przyjałkowski (1972, 1978) and Stefański (1965) were among the first to study the interactions between parasites and the intestinal flora in laboratory rodents. They found that the bacterial microflora positively influenced the development of nematodes, such as pinworm *Aspicularis tetraptera* and pork roundworm *Trichinella spiralis*. They showed that the natural bacterial flora, including *Bacteroidetes*, *Firmicutes* and *Mollicutes*, is indispensable for the successful invasion by these parasites [3,63,64]. However, in a study by Houser (1968) [65], the bacterial microbiota of the gastrointestinal tract did not influence the development and infection by *H. diminuta*, which may have been associated with the disturbances resulting from the intake of the enriched rat diet used in that experiment. The latest results show that experimental hymenolepidosis in rats is associated with changes in the number and composition of bacterial microflora [66], with a changed intestinal flora composition in the cecum in rats infected with *H. diminuta*, and a decreased percentage of *Bacillus* spp. in relation to *Clostridium* spp. [66]. On the other hand, a study by Wegener (2017) study showed small changes in the microbiota composition of female Wistar rats infected by *H. diminuta*. Taxonomic composition at the genus levels did not change even at the early stage of tapeworm infection, usually characterized by high immunogenicity [67]. The discrepancies in the results regarding the composition of the intestinal flora in hymenolepidosis may result from differences between the research methods used. The development of microbiology and new research techniques, including sequencing techniques, has supplanted old conventional methods that, although more accurate, still depend to some extent on the number of sequence errors introduced by the applied sequencing technology and bioinformatics used to analyze these sequences.

### 2.4. Changes in Ion Transport in the Host’s Digestive Tract

Mechanisms of glucose transport, ion transport such as Na^+^, K^+^ and Cl^−^, and water in the digestive tract, are very complex [68]. In the small intestine, the transport of water takes place by means of simple diffusion, and the absorption of electrolytes is performed, inter alia, via active transport. The basolateral membrane of the intestine is responsible for the absorption of Na^+^ via active transport, and the upper part of the small intestine is involved in the transport of Cl^−^ by passive diffusion [69]. As demonstrated, ion transport processes may be disturbed at the moment of stimulation of the immune system due to an infection with intestinal parasites [70,71]. The key role in these mechanisms is played by the lymphatic tissue found in the mucosa and submucosa of the gastrointestinal tract [72]. The effect on intestinal transport and motor activity is also exerted by mediators of inflammation: mast cells, macrophages, neutrophils and eosinophils released due to the presence of the pathogens [70]. An example of this is mast cell secretion of histamine, which affects the secretion of chloride ions and inhibits the absorption of NaCl in the gastrointestinal tract of the rat [73].

Since *H. diminuta* lives in the small intestine of the host, it interferes with the digestion and absorption in the host [35,70]. Research shows that *H. diminuta* can affect the absorption process by sequestering 3′,5-cyclic guanosine monophosphate (cGMP), which connects to the mucous membrane of the intestine via cGMP receptors, while altering intestinal motility and slowing down intestinal processes [74].

The transport of ions through the epithelium can be carried out by transcellular and extracellular pathways. Transcellular transport is targeted (dependent on the transport systems in cell membranes) and active (requires energy). The basic measure of the electrogenic transport of the ions is the transepithelial electrical potential (PD). The value of this parameter depends, inter alia, on the transport of sodium and chlorine ions through ion channels located in epithelial cells. In contrast, extracellular transport is bi-directional and passive. It occurs through gap junctions in the basolateral and in the apical part via tight junctions between epithelial cells [75]. The integrity of tight junctions and the degree of permeability of a given tissue for ions is determined by the measurement of transepithelial electrical resistance (R).

The transport of ions in the rat intestine and the impact of *H. diminuta* infection in this regard have not been fully understood. Researchers have reported the effect of other intestinal parasites on ion transport in the gastrointestinal tract, including the effect of *T. spiralis* infections on intestinal ion transport in the colon in the domestic guinea pig *Cavia porcellus*, and the effect of *F. hepatica* infection on ion transport in the rat intestine [76,77,78]. Research by Kosik-Bogacka et al. (2010, 2011) [70,71] showed that infection with *H. diminuta* causes changes in the transport of ions in the host’s small and large intestines. In the small intestine of rats, the value of transepithelial electrical potential (PD) had decreased at the 8th day post infection (dpi) with *H. diminuta*, where it remained until 60 dpi. Similarly, in the large intestine of rats infected with the tapeworm, a decrease in PD was observed, especially 60 days after *H. diminuta* infection. In the small and large intestines of tapeworm-infected rats, a decrease in occludin immunoexpression was observed, indicating that tight junctions were unsealed, which correlated with a decrease in transepithelial electrical resistance (R). On the basis of the immunohistochemical reaction, changes in the location and intensity of the reaction indicating the presence of occludin in the small and large intestine of rats infected with *H. diminuta* were found in comparison to the control group (not infected with tapeworms). In rats, starting from 8 to 40 dpi, occludin in enterocytes changed location from the point of contact of the cells to the apical or basal part of the cells, both in the small and large intestine. Electrophysiological studies showed a reduction in R in the small intestine, especially in rats from 25 to 60 dpi, compared to control rats. In the large intestine, R decreased only at 40 dpi [70,71].

The inhibition of ion transport and the reduction in the value of the transepithelial electrical potential by *H. diminuta* reflect the strategy and adaptation mechanisms used by the parasite. Avoiding and slowing the secretory processes, including the influence on ion transport in the host’s intestines, delay the removal of the parasite from the rat’s body. This process helps the tapeworm to achieve its goal; i.e., to reach maturity and produce offspring.

It has also been noticed that in the course of experimental hymenolepidosis in the rat there is a decrease in sensitivity to mechanical stimuli, where the use of mechanical stimulation and administration of capsaicin did not induce significant changes in transepithelial electrical potential in the small and large intestine of rats. In comparison, in animals of the control group, these stimuli did cause changes in ionic currents in the gastrointestinal epithelium. The reduction or complete lack of sensitivity to mechanical stimuli of the small and large intestinal epithelium rats infected with *H. diminuta* may be associated with decreased secretion of non-cholinergic transmitter (NANC) neuropeptides from the C-fiber endings. However, such a significant reduction in transepithelial electric potential difference in the bowels of rats infected with *H. diminuta* indicates the blockade of ion transport paths in the epithelium of the host’s gastrointestinal tract. It can be concluded that in the course of hymenolepidosis changes occur both in the active and passive extracellular ionic flow, induced by stimulation of nerve fibers (mainly C-fibers) and activation of inflammatory mediators. In addition, in the course of experimental hymenolepidosis in rats, the transport of sodium and chlorine ions in the small intestine and thick host epithelium are inhibited [70,71].

The preservation of intestinal integrity can also be assessed using various markers such as sugars (lactulose, mannitol, rhamnose), polyethylene glycols, and radioactive substances including ^99^-Tc-labeled-diethylenetriaminepentaacetic acid (^99m^Tc-DTPA) and ^51^-Cr-labeled-ethylenediaminetetraacetate (^51^Cr-EDTA). A study by Zimmerman (2001) examined the effect of ^51^Cr-EDTA on intestinal permeability at 6, 15, and 32 days after infection. An increase in intestinal permeability was observed only in the small intestine, at 15 and 32 days after the invasion, in comparison to non-infected rats. There were no significant differences in the jejunum, and changes that occurred in the small intestine did not recede after the removal of *H. diminuta* [74]. In contrast, Podesta and Mettrick (1974), in their experiment based on the conventional intestinal-loop technique, observed a decrease in intestinal permeability and decreased absorption of electrolytes and glucose in the small intestine of the rat during experimental hymenolepidosis [79].

**Figure 1:**
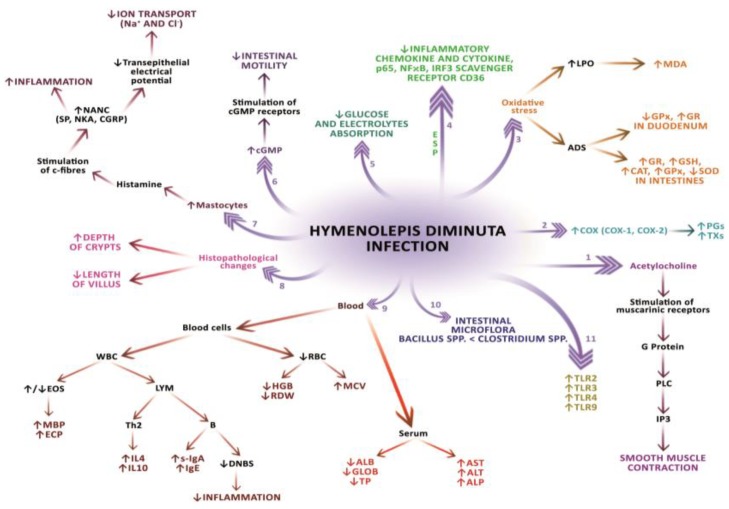
Biochemical and molecular mechanisms of *Hymenolepis diminuta*’s effect on the host organism. **1**. The parasite affects changes in the metabolism of acetylcholine, which stimulates the action of muscarinic receptors by changing their conformation. The muscarinic receptors can modulate the contraction by binding to the G protein. Activation of phospholipase C (PLC) by the G protein results in the formation of the inositol triphosphate signal molecule (IP3) and thus an increase in Ca^2+^ concentration in the cytoplasm. Ca^2+^ ions initiate a contraction, and an increase in their concentration is associated with the greater strength of contraction; **2**. Hymenolepidosis causes an increase in the activity and expression of COX-1 and COX-2 that produce TX and PG; **3**. *H. diminuta* in the host’s body may cause oxidative stress manifested by an increase in lipid peroxidation and their products, such as MDA (malondialdehyde dialdehyde), and changes in antioxidant enzyme activity in various parts of the gastrointestinal tract: in the duodenum, a decrease in GPx activity and increase in GR activity; in the intestine an increase in GR, GSH, CAT, and GPx activity and a decrease in SOD activity; **4**. The excretory-secretory products (ESP) of the parasite inhibit pro-inflammatory cytokines and chemokines, reducing the expression of transcription factors (NFκB, p65, IRF3) and the CD36 scavenger receptor; **5**. *H. diminuta* infection causes a reduced absorption of electrolytes and glucose in the small intestine of the host; **6**. By secretion of cGMP, which connects to the mucous membrane of the host intestine via the cGMP receptor, *H. diminuta* can affect intestinal peristalsis; **7**. In the course of hymenolepidosis, the host organism produces inflammation mediators, including mast cells. The histamine-secreted mastocytes stimulate the nervous system C-fibers that lead to the release of non-noradrenergic non-adrenaline (NANC) neuropeptides, including P (SP), neurokinin A (NKA), and calcitonin-related peptides (CGRP). This phenomenon may lead to neurogenic inflammation and may cause a reduction of transepithelial electrical potential, reducing ion transport; e.g., Na^+^ and Cl^−^; **8**. The parasite reduces villus length and deepens the crypts in the host’s digestive tract; **9**. *H. diminuta* causes disturbances in hematological blood values and changes in the composition of plasma: an increase in alanine aminotransferase (ALT) and aspartate aminotransferase (AST) and alkaline phosphatase (ALP), a decrease in biochemical parameters, including total protein (TP), albumin (ALB) and globulins (GLOB). Hymenolepidosis causes a reduction in the number of red blood cells (RBC), hemoglobin (HGB), red cell distribution width (RDW), and an increase in mean red cell volume (MCV). In the white blood cell system (WBC) there is a decrease in the number of cases or an increase in the number of eosinophils (EOS) that release the main alkaline protein (MBP) and eosinophil cationic protein (ECP). In the lymphocytic system, the activation of B lymphocytes responsible for the production of IgA class secreting antibodies (S-IgA) and IgE occur. In addition, B lymphocytes from *H. diminuta*–infected rats reduce the effects of dinitrobenzene sulfonic acid (DNBS), favoring the suppression of colitis and an increase in the production of interleukins (IL-4 and IL-10); **10**. Hymenolepidosis causes changes in the composition of the bacterial microbiota in the gut of the host in the form of reducing the percentage of *Bacillus* spp. in relation to *Clostridium* spp. **11** *H. diminuta* infection affects the growth of Toll-like receptor (TLR) expression, including TLR2, TLR3, TLR4 and TLR9 in the host’s digestive tract.

In conclusion, infection with *H. diminuta* causes a significant decrease in transepithelial electric potential difference, while at the same time blocks the transport of chloride and potassium ions and facilitates the movement of molecules in the ileum of the gastrointestinal tract (Figure 1). Mechanisms resulting from the effect of the parasite on the digestive system are related to the limitation of the secretory and excretory functions of the host.

### 2.5. Morphological and Biochemical Changes in the Host’s Blood

Parasitic infections, including intestinal colonization with *H. diminuta* may cause disturbances in hematological blood parameters and changes in plasma composition (Figure 1) [80,81,82].

Research by Goswami et al. (2011) showed that Wistar rats infected with *H. diminuta* showed reduced hemoglobin values (HGB), while other parameters, including the number of red blood cells (RBC), erythrocyte sedimentation rate (ESR), hematocrit (Hct), white blood cell count (WBC) and differential leukocyte count (DLC) were not significantly changed [16]. However, Kosik-Bogacka et al. (2010) observed that the number of red blood cells (RBC) and hemoglobin (HGB) decreased from 20 days post infection (dpi) and remained lower until 60 dpi. Red blood cell distribution with (RDW), a parameter determining the degree of anisocytosis (differences in the size of blood cells), was significantly smaller in relation to the control group. It was shown that RDW was negatively correlated with the duration of infection. The lower RDW suggests decreased anisopoikilocytosis. In addition, the decrease in RBC and HGB in infected rats in comparison with non-infected rats may suggest iron-deficiency anemia. In addition, there was a positive correlation between the duration of infection and the mean corpuscular volume (MCV) in blood from infected rats (macrocytosis). The study by Kosik-Bogacka et al. showed that *H. diminuta* infection may cause macrocytic anemia, possibly caused by the malabsorption of vitamin B12 and folic acid in the gastrointestinal tract of infected rats [70]. Gill (2007) also noted increased MCV in rats infected with *H. diminuta* and *Hymenolepis* (*Rodentolepis*) *nana* [80]. A decrease in the red blood cell parameters due to the presence of *H. diminuta* may be caused by its effect on absorption processes in the gastrointestinal tract. The host’s response to malnutrition due to parasitic invasion is associated with deficiencies of vitamin B12 or B6, folic acid, or iron. Anemia can be manifested in decreased RBC, MCV, and Hct, and is observed primarily in invasions by the tapeworm *D. latum*.

Parasitic infections may be associated with an increase in the number of monocytes, neutrophils, and eosinophilsare [81,82,83,84,85]. Numerous studies have shown that an increased number of eosinophils in the blood accompanies parasitic diseases and allergic reactions. This is confirmed by the results of the Gill (2007), in which rats infected with *H. diminuta* and *H. nana* showed a threefold increase in WBC compared to the control group [80]. In the case of experimental hymenolepidosis, however, Kosik-Bogacka et al. (2010) showed that the number of eosinophils (EOS) and their percentage in the total leukocyte count, gradually decreased in infected rats, starting from 8 dpi. Also, the percentage of basophils in the total amount of leukocytes had decreased at 8 dpi and 30 dpi. Therefore, it can be assumed that hymenolepidosis did not induce a strong immune response in the rat’s body. The observed low percentage of basophils was probably caused by a high white blood cell count compared to [70].

Biochemical parameters, including total protein, albumin and globulin were significantly reduced in wild rats infected with the tapeworms relative to the group of non-infected rats [80]. It was also found that the values of alkaline phosphatase, alanine and aspartate aminotransferases increased significantly in wild-type rats infected with *H. diminuta* compared to the control group of wild and Wistar rats, and in Wistar rats infected with *H. diminuta* [16]. Research on enzyme activity suggests that the parasite interacts not only at the site of infection, but also at distant organs, including the liver. Significantly elevated liver enzymes, such as an increase in alkaline phosphatase and aminotransferases, indicate liver dysfunction in rats infected with *H. diminuta* [16,83].

### 2.6. Changes in the Host’s Antioxidant Enzymes Activity

Parasites that live in the host’s body can cause an immediate hypersensitivity reaction and oxidative stress [86]. Generated in excess, reactive oxygen species (ROS) cause protein damage, DNA damage and lipid peroxidation, leading, inter alia, to impaired membrane integrity and changes in cellular metabolism [87,88]. Lipid peroxidation (LPO) can be an indicator of the intensity of oxidative stress in cells and tissues. The measurement of malondialdehyde concentration (MDA) resulting from the breakdown of polyunsaturated fatty acids is used as an indicator of lipid peroxidation production [89]. LPO products modify the properties of cell membranes, resulting in increased permeability of membranes to H^+^ ions and other substances, reducing electrical potential differences on both sides of the membrane [90]. An increase in the concentration of LPO and a decrease in the concentration of glutathione stimulates the enzymes of the metabolic pathway of arachidonic acid and increases the transport of calcium ions to the cells [91].

Reactive oxygen species can act directly on the intestinal mucosa by destroying the mucus, disrupting mucin synthesis and causing an increased disintegration [92]. Free radicals secreted by host eosinophils can directly damage parasites. Such reactions have been described in mice infected with dwarf tapeworm *H. nana* [93]. In addition, many researchers believe that ROS may participate directly or indirectly in the removal of tapeworms [94].

A study by Kosik-Bogacka et al. (2011) showed that infestation of rats with *H. diminuta* is accompanied by changes in the activity of antioxidant enzymes in various sections of the gastrointestinal tract, especially in the jejunum. A significant decrease in superoxide dismutase (SOD) activity was observed in all the examined fragments of the gastrointestinal tract compared to the control group, especially in the jejunum 60 days post infection. In addition, in the host from 8 dpi to 40 dpi there was a decrease in catalase (CAT) activity in the gastrointestinal tract, and at 60 dpi the activity of this enzyme increased. The study also found an increase in glutathione peroxidase (GPx) activity in the colon at 60 dpi. The above-mentioned changes in the activity of the enzyme that protects cells from the action of hydrogen peroxide (CAT and GPx) may have been caused by a higher demand for this enzyme due to increased H_2_O_2_ concentration in the intestinal lumen of the *H. diminuta*–infected rats. However, in the duodenum at 40 dpi, low GPx activity may have been caused by exhaustion of the antioxidative capacity of the body during the intensified inflammatory process. In the infected rats there was an increase in glutathione reductase (GR) activity in the duodenum and colon at 60 dpi and in the jejunum starting from 16 dpi. The study also showed no change in reduced glutathione (GSH) concentration in the duodenum and a significant increase in the jejunum and colon, respectively from 40 to 60 dpi and from 16 to 60 dpi. There was also a significant increase in LPO products in the duodenum and in the jejunum of rats infected with *H. diminuta*, whereas in the colon the concentration of LPO was similar to that in the control group [95].

In experimental hymenolepidosis there are changes in the activity of antioxidant enzymes and concentrations of non-enzymatic antioxidants, which may be at least partly due to oxidative stress as indicated by the increased concentration of lipid peroxidation products [95]. Skrzycki et al. (2011) showed that *H. diminuta* has developed adaptation mechanisms aimed at suppressing the host’s defense mechanisms by reducing the effects of oxidative stress. Tapeworms can counteract ROS activity by increasing the activity of antioxidant enzymes in various parts of their body [96].

Recent research shows that *H. diminuta* has evolved adaptive mechanisms that facilitate its development in the host organism, for example secretory capacity of immunogenic proteins and enzymatic systems responsible for protection against oxidative stress, including thioredoxin [11,97]. The proteomic analysis of two invasive forms, tapeworm cysticercoid and adult stages, by Sulima (2017), found a very large number of proteins responsible for the invasion, development, and adaptation of *H. diminuta*, including thioredoxin, at both stages of parasite development [97].

### 2.7. Changes in Cyclooxygenase Activity and Development of Inflammatory Reaction

Cyclooxygenase (COX) isoforms are detected in the central nervous system, gastrointestinal tract, circulatory system of the kidneys, vascular endothelium, placenta, heart, cartilage, and lungs, performing important functions in the metabolic regulation of tissues and organs, primarily in the digestive, circulatory, respiratory and nervous systems. Cyclooxygenases are involved in the metabolism of arachidonic acid to eicosanoids; including thromboxanes (TX), prostaglandins (PG) and prostacyclins (prostacyclin, PGI) [98]. In the gastrointestinal tract, the presence of cyclooxygenase 1 (COX-1) is found in most cells contributing to the production of PG under physiological conditions, whereas the expression of cyclooxygenase 2 (COX-2) is described in inflammatory processes induced by LPS, growth factors, ROS, and cytokines [99]. COX-1 is involved in maintaining homeostasis under physiological conditions and in early inflammatory conditions, causing the production of TXs and PGs, while COX-2 in late inflammation stages induces PG production [100].

COX-1 activity in the mucous membrane of the stomach and intestines in humans and rats is low [98]. Experimental studies show that COX-2 activity is also low in the gastrointestinal membrane of the rat under physiological conditions [101]. Thus, an increase in the activity of these enzymes can be used to monitor inflammation in the digestive tract. A study by Kosik-Bogacka et al., (2016) aimed to check the effect of *H. diminuta* infection on the transformation of arachidonic acid and the production of eiconosoids (PGs and TXs) in the colon and rat jejunum of rats. They determined that the expression and activity of COX-1 and COX-2 was responsible for the synthesis of prostaglandins and thromboxane. The study showed an increase in COX activity and expression in rats infected with *H. diminuta*, which could be due to increased levels of free radicals and weakened defensive antioxidative mechanisms [100]. Also, Ahmad et al., (2015) [102] found that hymenolepidosis in rats causes increased expression of cyclooxygenase 2 in the gut. In addition, that study showed increased expression of tumor necrosis factor (TNF-α) and increased induction of nitric oxide synthase.

Inflammation is associated with increased expression of cytokines, chemokines, and COX. In our study, a relationship was found between an increase in COX activity and *H. diminuta* infection. In view of the above, it can be assumed that the inflammation resulting from the *H. diminuta* infection increases the activity of COX and their products. Probably, the increased activity and expression of COX during hymenolepidosis is caused by the weakening of the antioxidant activity of the host caused by the presence of the parasite in the gut. In addition, tapeworm–induced inflammation may cause an increase in the level of free radicals resulting in the elevated COX activity.

### 2.8. Changes in the Expression of Toll-Like Receptors and the Mechanisms of Immune Response

Due to the direct contact of intestinal parasites with the mucous membrane of the digestive system, parasites affect the host’s immune system. A lack of integrity in the mucous membranes can lead to disturbances in homeostasis. The immune system of the digestive tract includes gut associated lymphoid tissue (GALT), with clusters of lymphoid follicles, single lymphoid papules, single lymphocytes, including B and T lymphocytes, macrophages, dendritic cells and proinflammatory cytokines [103]. The GALT barrier is a cylindrical epithelium with enterocytes, goblet cells and intraepithelial leukocytes and Paneth cells, as well as epithelial cell junctions: occluding junctions and adherens junctions [104,105]. In addition, factors that protect the digestive system against pathogens include low pH of the gastric juice, the proteolytic enzymes, lysozyme, antimicrobial peptides and physiological gastrointestinal flora [106,107].

In the case of helminth infections, host defense mechanisms are mediated by the immune response regulated by Th2 cells, in which eosinophils play a major role. The presence of intestinal parasites in the host organism also causes an increased synthesis of immunoglobulin E (IgE). Via the reaction of IgE with antigens on the surface of the parasite, the pro-inflammatory mediators are released from basophils, mast cells and eosinophils, causing inflammatory reactions. Then the stimulated Th2 lymphocytes can secrete interleukins such as IL-4, IL-5, and IL-13. In turn, IL-5 causes increased secretion of eosinophils that bind to the parasite, while releasing the main alkaline protein (MBP) and eosinophilic cationic protein (ECP). As a consequence, this mechanism causes damage to the parasite and its expulsion from the host organism [13,108]. The defense of host mucous membranes against a parasitic invasion is mainly caused by the activation of IgA class antibodies: secretory IgA (S-IgA), whose receptors are found on monocytes, neutrophils, eosinophils and phagocytic cells. The role of these antibodies is to uptake antigens and prevent them from penetrating through the mucous membranes [6].

Helminths and their excretory-secretory products (ESP) can suppress the immune response and influence inflammation to avoid host defense mechanisms. Numerous studies indicate the key contribution of macrophages to the host immune response during parasitic invasion. Phagocytic cells are modulators and effector cells of the immune system. Two macrophage phenotypes, M1 and M2, can be distinguished according to the type of polarization. M1 are macrophages activated by Th1 cells and cytokines, whereas M2 are activated alternatively, depending on the type of activation signals. Dendritic cells and macrophages belong to the first cells of the immune system that affect the course of parasitosis. The adaptation mechanisms of tapeworms consist in the production of surface proteins and secretion of excretory secretory products (ESP), which may have influence on the type of macrophage polarization. Zawistowska-Deniziak (2017) showed that *H. diminuta* and ESP have an effect on human THP-1 macrophages by stimulating the secretion of cytokines and inflammatory chemokines derived from macrophages. In that study, ESP were observed to reduce the expression of cytokines (IL-1α, TNFα, TGFβ and IL-10) and chemokines (IL-8, MIP-1α, RANTES (regulated on activation, normal T-cell expression and secreted/CCL5) and increase the expression of s-ICAM (intercellular adhesive molecule-1) and chemokine CXCL10. In addition, macrophages activated by *H. diminuta* showed increased expression of inflammatory factors. The tapeworm and ESP were shown to have an effect on the ERK1/2 (extracellular signaling kinase) phoshorylation pathways, STAT2, and STAT3 (signal transduction and activation of transcription), AMPKα1 (AMP-activated protein kinase) and Hsp60 (heat shock protein) [109]. Zawistowska-Deniziak (2017) suggested that exposure to *H. diminuta* antigens and ESP induces a mixed type of macrophage polarization. A similar study was conducted by Johnston (2010), who observed decreased *E. coli* LPS-induced production of TNF-α by macrophages as a result of exposure to the high-molecular extract of *H. diminuta* (HdHMW) [12]. Aira et al. (2017) showed that *H. diminuta* antigens induce M2 macrophage polarization and may reduce the maturation of *Mycobacterium tuberculosis* phagosomes in human monocyte-derived macrophages (hMDMs), induce early secretion of pro-inflammatory cytokines, and induce late anti-inflammatory response with increased IL-10 secretion [110]. In addition, Aira et al. (2017) showed that tapeworms intensify the action of *M. tuberculosis* by increasing the secretion and expression of CD163 [110]. This confirms previous assumptions about the possible stimulation of the host’s immune system against *H. diminuta* antigens. Adaptive mechanisms of the parasite stimulate antiinflammatory pathways in the host, thus avoiding expulsion of the tapeworm.

Research on the impact of *H. diminuta* antigens and its ESP on the host immune system has been conducted for many years. It was found that the mutual relations in the parasite-host system have been modified during the evolution. The parasites acquired new genes to avoid the immune response of the host. This transformation can be studied thanks to complex genomic and proteomic techniques. Proteomic analysis of antigens allows identification of *H. diminuta* proteins during the infection, providing information on individual stages of the tapeworm’s life cycle [27,97]. A study conducted by Sulima et al. (2017) describes the *H. diminuta* somatic antigens in the body of the definitive host. Researchers identified 70 antigenic proteins using classical two-dimensional gel electrophoresis (2DE) and immunoblotting techniques. Most of them were structural proteins (components of the cytoskeleton, muscle, and intracellular matrix), heat shock proteins (Hsp60, Hsp70, Hsp20, Hsp3 and sHsp), proteins involved in metabolic processes and enzymes belonging to the family of cysteine proteases. The results of proteomic analysis showed that *H. diminuta* cysticercoid proteins include proteins involved in the mechanisms of bypassing/modulating the host’s immune system (e.g., actin) whose function is to stimulate ATP hydrolysis, myosin exhibiting ATPase activity and actin binding capacity, paramyosin, which modulates the host’s immune response, calpain involved in cellular proteolysis, and the HSP family proteins Hsp60 and Hsp70, which induce Treg and Hsp70 cells that are associated with immunomodulatory activity. In addition, researchers identified molecules involved in metabolic processes (i.e., phosphoenolpyruvate carboxykinase (PEPCK)), which are directly involved in hormonal and excretory systems, as well as carbohydrate metabolism; glucose-regulated 78kDa protein (GRP-78); and apolipoprotein AI (ApoA-I). Sulima et al. (2017) have also identified RBBP (retinoblastoma–binding protein) proteins, which are classified as cancer proteins. Data on the presence and expression of RBBP may be used in the future to develop antiRBBP therapies in host cancer transformation mechanisms. Many of *H. diminuta* proteins are associated with the host’s immune response to infections. However, in order to clarify the role of the individual proteins of *H. diminuta*, experimental studies should be extended to all stages of the tapeworm life cycle using the analysis of proteomes, secretomes (secretory proteins), and exosomes in a multidisciplinary approach [27,97].

Further research on proteins affecting the parasite-host system may provide new information on the pathogenicity of the rat tapeworm and allow the development of new treatments for inflammatory diseases. The most recent research concerns the use of immunological reactions in hymenolepidosis to inhibit inflammation of the distal part of the large intestine and rheumatoid arthritis in a mouse model [12,111,112,113,114,115]. The process of colitis is associated with an increased amount of monocytes in the blood and the activation of cytokine-secreting macrophages. Pro-inflammatory cytokines such as IL-1, -2, -6, -8, -12, -17, -23, -25, tumor necrosis factor (TNF) and interferon (IFN) induce and sustain inflammation. The process also involves the secretion of cytokines with antagonistic activity to pro-inflammatory cytokines—IL-4, IL-10 and IL-13, which inhibit inflammatory processes in the large intestine [116]. Numerous reports describe the mechanisms of influence of *H. diminuta* on the production of interleukins in colitis. Melon (2010), Arai (2018) and Johnston (2010) have shown the inhibition of dinitrobenzenesulfonic acid–induced colitis (DNBS) by increased production of IL-4 and IL-10 in mice infected with rat tapeworm [12,112,113]. In addition, Johnston (2010) observed decreased TNF-α secretion in mice infected with *H. diminuta* [12]. A study by Wang (2005) also showed changes in the secretion of inflammatory cytokines by stimulation with *H. diminuta* extract. Wang (2010) observed that immune cells derived from human blood and spleen increased the production of IL-12, IL-10 and IFN-γ, and reduced the secretion of IL-2 and IL-4 [117]. Reyes (2015) showed that CD19+ lymphocytes from *H. diminuta*–infected mice reduced the effects of DNBS, and thus promoted the suppression of colitis [115]. In turn, Arai et al. (2018) showed that the *H. diminuta* antigen inducing an immune response in previously infected mice could be used to limit the severity of colitis regardless of the age of the host [118]. Graepel (2013) [111] found an exacerbation of the disease as a result of *H. diminuta* infection in a mouse with rheumatoid arthritis. The experiment showed an increase in the level of factors responsible for the development of inflammation of activation, including C5a and mast cells. In contrast, McKay (2009) in an experiment on rats with hymenolepidosis and stimulated with acetic acid to produce lesions in the gastric mucosa did not show a mitigating effect of the tapeworm on the resulting ulcer [119]. Zawistowska-Diniziak et al., (2017) [109] showed that *H. diminuta* and its excretory–secretory products (ESP) have an inhibitory effect on proinflammatory cytokines and chemokines, and also reduce the expression of transcription factors (NFκB, p65, IRF3) and scavenger receptor CD36, involved in many processes of innate immunity, including removing dead cells and the process of inhibiting tumor angiogenesis. Reyes (2016) [114], on the other hand, showed that IL-22 reduces the anti-parasitic response by inhibiting IL-25, and also limits the inhibition of colitis during hymenolepidosis. Smyth et al. (2017) conducted research using *H. diminuta* cysticercoids (HDC) as potential agents to alleviate inflammation in the host’s digestive tract. The antiinflammatory HDC therapy employs immunomodulation mechanisms of the host immune system. The authors stated that the efficiency of this helminthic therapy depends on many factors, including the patient’s individual characteristics and the purpose of therapy. It was also noted that HDC could be used to treat migraine headaches, anxiety disorders, depression, and Tourette’s syndrome. HDC therapy was also shown to be effective in autoimmune diseases including multiple sclerosis and non-specific inflammatory bowel diseases. However, the use of HDC in the treatment of chronic diseases is associated with the risk of complications including gastrointestinal diseases, irritable bowel syndrome (IBS) and inflammatory bowel diseases (IBD). This risk and a small number of clinical trials make it necessary to conduct further research of the effect of HDC on the host [120].

**Figure 2:**
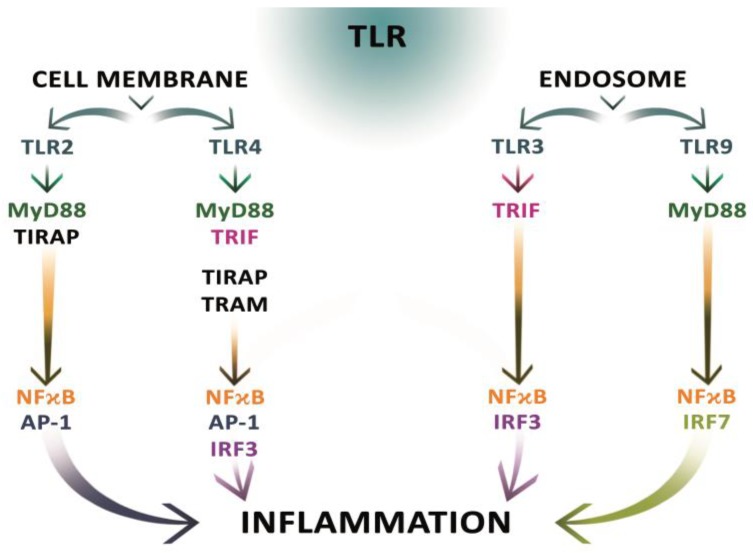
Signaling pathways for TLR2, TLR3, TLR4, and TLR9. Signaling pathways derived from TLRs are very complex. Two major pathways are suggested: MYD88 (myeloid differentiation primary response gene 88) and TRIF (TIR-domain containing adapter inducing IFN-ß). MYD88-dependent signaling pathways are derived from TLR2, TLR4, and TLR9, whereas TLR3 uses TRIF protein for transduction. To activate MYD88, TLR4 uses TIRAP (TIR-domain containing adapter protein) and TRAM (TRIF-related adapter molecule), while TLR uses TIRAP only. As a result of MYD88–dependent signaling pathways, transcription factors are activated: AP-1 (activator protein 1) and NFκB (nuclear factor kappa-light-chain-enhancer of activated B cells), while transcription factors within the MYD88–independent pathways activate IRF (IFN regulators factor) 3 and 7 and NFκB. TLR4 is a receptor that has signal transduction capabilities in both these pathways [138,139].

Toll-like receptors (TLR) play an important role in immunity. They recognize pathogen associated molecular patterns (PAMP) and induce the synthesis of antibacterial and antifungal proteins. Biochemical studies have shown that the TLR are transmembrane proteins, members of the type I receptor for the IL-i 1 interleukin. TLR2 and TLR4 are the best-known Toll–i like receptors. Based on experimental studies, it was found that the ligands for TLR2 are the products of decay of dead cells [121], and for TLR4 these are heat shock proteins [122]. TLR are found mainly on cells of the immune system, inter alia on dendritic cells, monocytes, macrophages, B lymphocytes, mast cells, eosinophils and neutrophils [123]. In the gastrointestinal wall, different types of TLR are found in immunologically reactive sites with specific functions: TLR2 and TLR4 are located in the intestinal crypts, and TLR3 and TLR9 inside intestinal epithelial cells [124] (Figure 2). In the crypts of the intestine TLR2 is responsible for the recognition of peptidoglycan and bacterial lipoproteins, and TLR4 is responsible for the diagnosis of LPS (lipopolysaccharides) and lipotichichoic acid. TLR3, which binds dsDNA, participates in the diagnosis of viral infections, while TLR9 binds to unmethylated CpG DNA dinucleotides [103,125,126,127,128,129,130,131,132,133]. The presence of TLR allows for the early elimination of pathogens. They play a role in initiating inflammatory processes and repairing damaged tissues [134].

Many papers deal with the role of TLR in initiating an immune response in bacterial infections. There are far fewer publications regarding the role of TLR in response to parasitic infections. There is also little data on the role of TLR in the innate and acquired responses of the host immune system during hymenolepidosis. The first research describing these mechanisms was carried out by Kosik-Bogacka et al. (2012). That study concerned the role of Toll-like receptors in the pathomechanism of hymenolepidosis, and more specifically the expression of TLR2, TLR3, TLR4, and TLR9 in the gastrointestinal tract of control rats and *H. diminuta* infected tapeworms using RT-PCR (reverse transcription and polymerase chain reaction) and immunohistochemistry. Using molecular and immunohistochemical methods, the control rats showed differences in the expression of TLR2 and TLR4 between the small and large intestines. Expression of these receptors was greater in the large intestine than in the small intestine. This could be due to differences in the composition of the intestinal bacterial flora in various parts of the gastrointestinal tract [135,136]. In rats infected with *H. diminuta*, those authors found an increase in TLR2 and TLR4 expression compared to the control group, especially in the small intestine. The increase in TLR2 expression in the small intestine was found in rats 6 and 8 days post *H. diminuta* infection, while TLR4 at 4 dpi, 6 dpi, and 8 dpi. In the large intestine, an increase in TLR2 and TLR4 expression was found in 8 dpi and 6 dpi rats, respectively [135]. Analysis of immunohistochemical reactions showed significant changes in the location of TLR2 and TLR4 in the gastrointestinal tract at 4 dpi and 8 dpi compared to the control group. In the small intestine of control rats, TLR2 and TLR4 were located in the basal portion of absorption cells (enterocytes). In contrast, in rats infected with tapeworms (4 dpi and 8 dpi) TLR2 and TLR4 were located across the entire height of the some absorption cells. In the large intestine of the control rats TLR2 and TLR4 were located around the nucleus of the absorption and goblet cells, and in infected rats (4 dpi and 8 dpi), these receptors were observed in the cytoplasm under and over the nucleus in the absorption cells. The intensity of the positive reaction increased with subsequent days after infection with a tapeworm [135,136]. Changes in the expression level of TLR2 and TLR4 in the small and large intestine of rats infected with *H. diminuta* may have been induced by tapeworms and glycolipids [137]. In addition, the existence of the parasite in the small intestine of the host could cause changes in the composition and amount of the bacterial intestinal flora.

In the case of the analysis of TLR3 and TLR9 expression, Kosik-Bogacka et al., (2014) did not report any significant differences in the expression of these receptors in the control group between the small and large intestine. In contrast, in the study group, an increase in the expression of mRNA and TLR3 receptor protein was observed in the small intestine of rats 16 days post *H. diminuta* infection and 16 dpi and 25 dpi for TLR9 in comparison to the control group. Those authors also found an increase in TLR3 and TLR9 expression in the large intestine at 16 dpi, 25 dpi, and 40 dpi. The results of the immunohistochemical reactions showed changes in TLR3 and TLR9 distribution in the small and large intestine at 16 dpi, 25 dpi, 40 dpi, and 60 dpi compared to the group of non-infected rats. The intensity of positive reaction for TLR3 was strongest at 16 dpi and TLR9 at 25 dpi. In the following days the expression of these receptors was smaller and smaller. In the control group in the small intestine TLR3 and TLR9 were distributed in the vicinal portion and on the apical surface of the absorption cells. In contrast, in the infected rats, TLR3 was located mainly around the nuclei of the absorbing cells and under the small intestine microvilli, and TLR9 was scattered over the entire height of the absorption cell. In the case of the large intestine in the control group, the expression of TLR3 was the strongest in the apical part of absorption cells and in the infected rats in the basal part. TLR9 in the large intestine in the control group was placed near the nucleus of the absorption cells, and in the infected rats they were distributed throughout their entire height [140].

Observed changes in the expression of TLR2, TLR3, TLR4, and TLR9 in the small and large intestine of rats infected with *H. diminuta* testify to the role of these receptors in the pathomechanism of hymenolepidosis [135]. It can be concluded that infection with *H. diminuta* is related to an immune response, and adaptation of the host organism to the antigens of this parasite and the course of comorbidities cannot be ruled out.

All the mechanisms presented in Figure 1 are described in this article.

## 3. Conclusions and Further Research Prospects

The molecular mechanisms known to date involved in the parasite–host system provide valuable information on the pathogenesis and factors affecting the course of hymenolepidosis. However, the multidimensional mechanisms described in this paper only to a small extent allow us to understand the interaction between the parasite *H. diminuta* and the host *Rattus norvegicus*. Previous studies show that *H. diminuta* infection affects the host organism, modulating its functioning. The tapeworm’s adaptive mechanisms had an effect on the processes of digestion in the host’s digestive tract, limiting its elimination, the host’s immunological reactions, and the activity of the antioxidant enzymes, causing inflammation and changes in blood hematological and biochemical parameters. In order to understand all the processes of the parasitic invasion and to address many unanswered questions, further research is required to describe the molecular mechanisms and pathways of the immune response. Deepening the knowledge about interactions in the parasite–host system will enable understanding the parasitic invasion at all stages of the tapeworm life cycle and the modulation of the host’s immune system. This requires further multidisciplinary research using proteomic techniques and expanding the scope to include secretomes and exosomes.

Due to the fact that *H. diminuta* infection affects a number of reactions occurring in the host organism, it can be assumed that it may also be involved in mechanisms other than those discussed in the presented works; e.g., the apoptosis of intestinal epithelial cells. From current literature it appears that an understanding of the metabolic pathways and regulation of the immune response in hymenolepidosis may be helpful in establishing the mechanisms of allergic and inflammatory diseases. It may also lead to the discovery of new treatments for inflammatory diseases using HDC.
